# Structure and Thermoelectric Properties of Bi_2−*x*_Sb*_x_*Te_3_ Nanowires Grown in Flexible Nanoporous Polycarbonate Templates

**DOI:** 10.3390/ma10050553

**Published:** 2017-05-19

**Authors:** Anuja Datta, Abhijeet Sangle, Nick Hardingham, Charles Cooper, Max Kraan, David Ritchie, Vijay Narayan, Sohini Kar-Narayan

**Affiliations:** 1 Department of Materials Science & Metallurgy, University of Cambridge, Cambridge CB3 0FS, UK; ad819@cam.ac.uk (A.D.); as2174@cam.ac.uk (A.S.); nick.hardingham@gmail.com (N.H.); maxkraan1@gmail.com (M.K.); 2 Cavendish Laboratory, Department of Physics, University of Cambridge, Cambridge CB3 0HE, UK; charles.f.cooper@gmail.com (C.C.); dar11@cam.ac.uk (D.R.)

**Keywords:** Bi_2−*x*_Sb*_x_*Te_3_, thermoelectric, electrodeposition, nanowires, Seebeck coefficient

## Abstract

We report the room-temperature growth of vertically aligned ternary Bi_2−*x*_Sb*_x_*Te_3_ nanowires of diameter ~200 nm and length ~12 µm, within flexible track-etched nanoporous polycarbonate (PC) templates via a one-step electrodeposition process. Bi_2−*x*_Sb*_x_*Te_3_ nanowires with compositions spanning the entire range from pure Bi_2_Te_3_ (*x* = 0) to pure Sb_2_Te_3_ (*x* = 2) were systematically grown within the nanoporous channels of PC templates from a tartaric–nitric acid based electrolyte, at the end of which highly crystalline nanowires of uniform composition were obtained. Compositional analysis showed that the Sb concentration could be tuned by simply varying the electrolyte composition without any need for further annealing of the samples. Thermoelectric properties of the Bi_2−*x*_Sb*_x_*Te_3_ nanowires were measured using a standardized bespoke setup while they were still embedded within the flexible PC templates.

## 1. Introduction

The rise of wireless sensing and communication technology in consumer electronics, health care and industry has led to an increased demand for wireless devices that are able to sustain themselves using ambient energy sources such as heat and vibrations. Thermoelectric generators (TEGs), without requiring any moving parts, can convert a temperature difference directly into an electrical current, and are therefore attracting widespread interest in generating power by recovering waste heat energy [1]. Thermoelectric (TE) materials therefore hold a lot of promise in power generation, via the Seebeck effect, as well as solid-state cooling via the converse Peltier effect [2]. The TE dimensionless figure-of-merit of a material at a temperature *T*, defined as *ZT* = *S*^2^*σT*/*κ* (where *S* is the Seebeck coefficient, *σ* is the electrical conductivity *κ* is the thermal conductivity), is essentially dependent on the material’s intrinsic electrical and thermal properties. So far, considerable efforts have been made toward enhancing the figure-of-merit values in other existing TE material classes, including tellurides [3,4,5,6], half-Heuslers [7,8], and silicides [9,10]. Also *ZT* can be often largely improved by nanostructuring [11,12,13]. In this respect, TE nanomaterials offer the scope for higher *ZT* values and simultaneously allow for miniaturization of TE devices required for small-scale energy harvesting technologies. Additionally, there is a considerable interest in developing flexible and lightweight TE materials and devices for thermal energy harvesting for a range of different applications. Here we have employed a simple and cost-effective one-step electrodeposition growth process to fabricate vertically aligned nanowires of the ternary compound, Bi_2−*x*_Sb*_x_*Te_3_, embedded within a flexible nanoporous PC template, where the effect of composition variation on the Seebeck coefficient is investigated.

Mixed ternary compounds of isostructural Bi_2_Te_3_ and Sb_2_Te_3_ (Bi_2−*x*_Sb*_x_*Te_3_ where *x* = 0 to 2) are among the most widely studied TE materials with a room temperature *ZT* value of ~1 [14,15,16]. A large variation in this alloy composition is possible without greatly affecting *ZT* when a precise doping is maintained, and hence this has been a focus for TE research. The crystal structures of Bi_2−*x*_Sb*_x_*Te_3_ TE alloys show anisotropy originating from the rhombohedral structure composed of quintuple atomic layer series along the c-axis, and therefore one-dimensional nano-confinement in this material may significantly affect the electronic and thermal properties [11,12,13]. Significant progress in enhancing *ZT* in Bi_2_Te_3_-based materials has been made through utilizing scalable processing techniques that can lead to nanostructuring [16,17,18,19], in order to achieve the desired shape, size, and other physical attributes that are useful controlling factors for enhanced TE performance. To this end, Bi_2−*x*_Sb*_x_*Te_3_ nanowires (NWs) have been extensively investigated, and TE performance of these alloy NWs are found to be improved due to the phonon scattering effects across the grain boundaries, and by manipulation of the band structure due to dimensional confinement [20,21,22,23]. In this regard, a solution-based synthesis processes such as electrodeposition is a widely used protocol for preparing NWs within porous templates and has been reported as a cost-effective and scalable method to grow Bi_2_Te_3_ based NWs with high density and large aspect ratios [24,25,26,27]. These NWs have been predominantly synthesized by a number of template-assisted methods using track-etched membranes [28,29], diblock copolymers [30,31], or anodized aluminum oxide (AAO) templates [24]. However, high thermal conductivities of the template matrices have been known to induce large thermal leakages [32,33]. Therefore, it is crucial to prepare TE NWs in flexible and robust polymer templates that have very low thermal conductivities themselves, and that provide additional mechanical support and protection to the embedded NWs. 

Our work therefore focuses on optimizing the growth parameters for different stoichiometries of ternary Bi_2−*x*_Sb*_x_*Te_3_ (*x* = 0, 0.5, 1, 1.5, 2) NWs of diameter ~200 nm and length ~12 µm in track-etched nanoporous polycarbonate (PC) membranes at room temperature, which was previously not been systematically studied. Controlling the doping concentration in the deposition solution leads to the variation in the stoichiometry in these high aspect ratio NWs. The resulting NWs were found to be highly crystalline and preferentially aligned with uniform compositional homogeneity. Furthermore, the high mechanical stability of the lightweight PC templates enabled the in-situ measurement of the thermoelectric properties of Bi_2−*x*_Sb*_x_*Te_3_ NWs, while they were still embedded and vertically aligned within the template. The work is of interest in the context of research toward fabricating flexible inorganic-organic hybrid TEGs. The synthesis process may be modified and extended to design NWs of other relevant Bi-based TE materials such as Bi and Bi*_x_*Sb*_y_* alloys with related crystal structures.

## 2. Materials and Methods

### 2.1. Template Preparation

**Figure 1:**
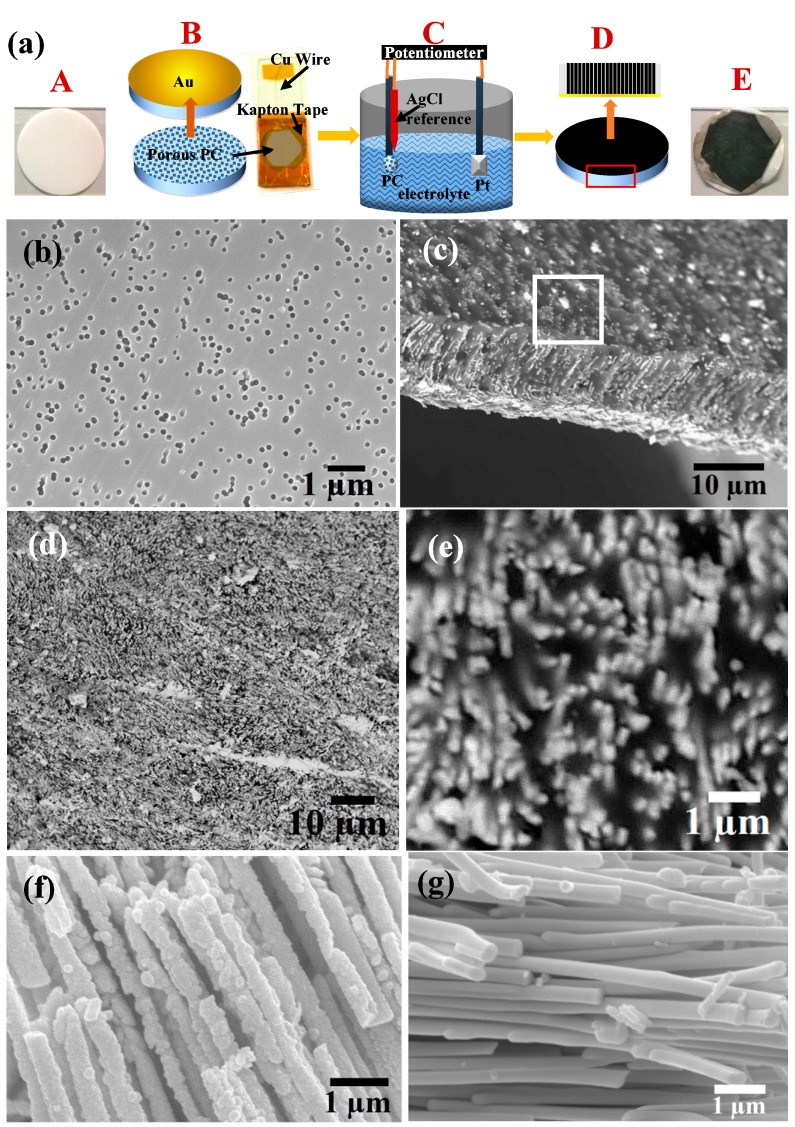
(**a**) Schematic showing the electrodeposition growth process of Bi_2−*x*_Sb*_x_*Te_3_ NWs inside polymer PC templates. Panel A is the original PC template and panel E is the template after deposition containing NWs; (**b**,**c**) are the SEM images of nanoporous PC templates before and after NW growth; (**d**) shows the surface of the NW filled template as indicated in (**c**) (white square); (**e**) Close-up view of the NW arrays inside the PC template; (**f**) Sb-rich Bi_2−*x*_Sb*_x_*Te_3_ NWs after dissolving PC template which show rough surfaces as compared to the Bi-rich NWs as shown in (**g**) which are smooth textured.

Nanoporous polycarbonate (PC) templates of thickness ~12 µm thick and having pore diameters ~200 nm with a pore density of ~25% (Whatman^®^ Cyclopore^®^, GE Healthcare, Wilkes-Barre, PA, USA) were used for growing the Bi_2−*x*_Sb*_x_*Te_3_ NWs (Panel A in Figure 1a). Prior to electrodeposition, the templates were coated on one-side with a layer of 100 nm thick Au layer by sputtering (Emitech^®^ K550, Quorum Technologies Limited, Lewes, UK) (Panel B in Figure 1a). The Au layer serves as both nucleation sites and electrode contact for the growth of NWs inside the porous template during electrodeposition [34]. A glass slide coated with Au was then used to attach the Au-coated template secured with Kapton tape, and a copper wire was soldered to the template for electrical contact to the template during the electrodeposition process (Panel C in Figure 1a).

### 2.2. Electrolyte Preparation

All the chemicals obtained from Sigma Aldrich (Cheshire, UK) were of 99.99 % purity, ACS grade and were used without further purification. To grow Bi_2−*x*_Sb*_x_*Te_3_ NWs, acidic aqueous electrolyte solutions were prepared. To achieve well-dispersed electrolytes, initially 1.5 mmol ground tellurium (Te) powder was dissolved in 6.4 mL 70% HNO_3_ at 60 °C and the solution was stirred using a magnetic stirrer, followed by addition of 10 mL DI-water. When Te was fully dissolved, required molar ratios (for *x* = 0.25, 0.5 and 0.75) of hydrated bismuth nitrate (Bi(NO_3_)_3_·5H_2_O)) was added at 60 °C. After bismuth nitrate was dissolved, 100 mL H_2_O was added and the solution was cooled down to room temperature. 1–5 gm of tartaric acid was added depending on the molar ratio of Sb required, which was dissolved at room temperature. The tartaric acid served to dissolve the antimony acetate added in the next step and prevented formation of unwanted Sb_2_O_3_ from antimony (III) acetate (Sb(CH_3_CO_2_)_3_) that was added in the end and dissolved at room temperature. The final solution was stirred at room temperature until a clear electrolyte mixture of Bi, Sb and Te precursors were obtained without any visible precipitants at pH ≈ 2.

### 2.3. Electrodepositon Process

The electrodeposition rig was set up in a three-probe configuration, where a Pt foil (20 mm × 20 mm) electrode was used as the cathode, while the electrode PC template and a Sigma Aldrich Ag/AgCl reference electrode served as the anode (Panel C in Figure 1a). A Versastat4^®^ from Princeton Applied Sciences was used to set up the potential difference and the data was recorded in VersaStudio^®^ [34,35]. Chronoamperometric electrodeposition (potentiostatic) was used where the potential difference and deposition time were varied and optimized to reach the optimum growth of the NWs and achieve maximum fill within the nanoporous PC templates (Panel D in Figure 1a). Under optimized conditions, −0.5 V was applied between the working electrode and the double junction Ag/AgCl reference electrode for ~90 min. The deposition was paused in the middle and the template surface was treated with stream of DI water to remove the powdery deposition on the template surface and the process was continued thereafter. When this intermediate cleaning step was not undertaken, the deposition residue blocked the nano-pores and incomplete growth of nanowires was observed (see Supporting Information). The solution was stirred throughout the deposition to avoid precipitation during the electrodeposition process. Finally, Bi_2−*x*_Sb*_x_*Te_3_ NW-filled PC templates were obtained (Panel E in Figure 1a). The PC template filled up with the NWs appeared to be black due to the presence of Bi_2−*x*_Sb*_x_*Te_3_ NWs inside, as shown in Panel E in Figure 1a).

### 2.4. Characterization

The phase and crystallinity of the Bi_2−*x*_Sb*_x_*Te_3_ NWs were characterized by X-ray diffraction (XRD) with a Bruker D8 diffractometer (Bruker AXS, Madison, WI, USA) equipped with Lynx Eye position-sensitive detector using Cu Kα radiation (λ = 1.5418 Åu) at room temperature. Peak shifts due to sample misalignment were adjusted while performing the XRD scans and background correction was taken care of by using a zero diffraction silicon substrate for mounting. Microstructural studies were carried out by field–emission scanning electron microscopy (FE–SEM, FEI Nova NanoSEM, FEI, USA) and transmission electron microscopy (HRTEM, JEOL 4000EXII, JEOL, Tokyo, Japan). For TEM analysis Bi_2−*x*_Sb*_x_*Te_3_ NWs were released and collected by dissolving the PC templates in chlorobenzene (99%, Sigma Aldrich) followed by centrifugation. The NWs were then dispersed in anhydrous ethanol and drop-cast onto TEM grids (Ted Pella Inc., Redding, CA, USA). Energy Dispersive X-ray Analysis (EDX, Oxford Instruments, Oxfordshire, UK) was carried out to map the elemental distribution and to scan the elemental distribution along individual Bi_2−*x*_Sb*_x_*Te_3_ NWs. Structural studies such as XRD and SEM were also carried out on the NWs freed from the template.

### 2.5. Thermoelectric Property Measurements

The electrical conductivities and Seebeck coefficients (relative to the electrodes and contacts) of the samples were measured by a bespoke measurement setup (see Supporting Information). It consisted of a pair of commercial Peltier cooler/heater elements (Multicomp^®^, 4.5 W, Farnell, UK) connected in series but with reverse polarity. A DC source was used to power the Peltier elements so that when one was heated the other was cooled and vice-versa. Both the Peltier elements were connected to heat reservoirs in the form of metallic blocks to ensure steady temperatures at a given DC bias powering the Peltier elements. The sample to be measured was sandwiched between the two Peltier elements to create a stable thermal gradient across the sample. Enamelled copper conducting wires were attached using Ag paint to the top and bottom faces of the sample in a pseudo four probe configuration. The Seebeck voltage and the electrical resistance of the sample were measured using a Keithley digital multimeter. The temperatures of the two Peltier elements were monitored by using an aerosol-jet printed Ag-track resistance thermometer [36], and sputter-coated Pd thin-film resistance thermometer, which were both calibrated against a commercial (Pt-100) platinum resistance thermometer (see Supporting information). A thermal grease (Servisol^®^ heat sink compound, Farnell, UK) was used to ensure good thermal contact between the Peltier elements and the sample.

## 3. Results and Discussion

The step by step reactions (inequilibrium) responsible for electrolytic deposition of Bi_2−*x*_Sb_*x*_Te_3_ NWs in acidic solutions can be described as:

3Te + 12HNO_3_ → 3HTeO_2_^2+^ (aq) + 12NO_2_ + 3H_2_O + 3OH^−^ (1)

2Bi(NO_3_)_3_(5H_2_O) (aq) + 6H_3_O^+^ → 2Bi^3+^ + 6HNO_3_ + 16H_2_O (2)

2Sb(CH_3_CO_2_)_3_ + 3C_4_O_6_H_4_ → 3Sb^2+^ + 6CH_3_COOH + 3C_4_O_6_H^2−^ (3)

3HTeO_2_^2+^ + Bi_2−*x*_^3+^ + Sb_*x*_^2+^ + 20 e^−^ + 9H^+^ → Bi_2−*x*_Sb*_x_*Te_3_ + 6H_2_O (4)

However, the actual mechanisms of reaction are far more complex [37], as the pH value of the electrolytic solution as well as the electrochemical potential has an influence on the crystallinity and stoichiometry of the NWs grown. The actual composition of Bi_2−*x*_Sb*_x_*Te_3_ NWs grown in PC controls their microstructure, crystallinity, and eventually thermoelectric behavior as shown in Figure 1b–g. Figure 1b shows the SEM image of the bare PC template indicating ~20% porosity, while Figure 1c) shows the Bi_2−*x*_Sb*_x_*Te_3_ NW-filled template. The representative cross sectional image of a torn-up PC template shown in Figure 1c reveals the growth of Bi_2−*x*_Sb*_x_*Te_3_ NWs, filling the template all through. Typical top surface of the Bi_2−*x*_Sb*_x_*Te_3_ NW-filled PC template is indicated by a square in Figure 1c and shown in Figure 1d). Profuse growth of NWs with protruding tips are clearly evident after the electrodeposition of Bi_2−*x*_Sb*_x_*Te_3_ NWs showing almost 100 % coverage. Bi_2−*x*_Sb*_x_*Te_3_ NWs of Sb-rich and Bi-rich compositions (nominal at %) were also released after dissolving the PC template (see experimental details) which are shown in Figure 1f,g, respectively. In the case of NWs, which are Sb-rich in composition, rough surface morphology in the NWs was observed. These NWs appeared to be rough, consisting of many nano-sized grains indicating poor crystallinity, while the Bi-rich NWs appear to have smooth, well-defined facets and hence, better crystallinity. This is further confirmed from XRD studies.

**Figure 2:**
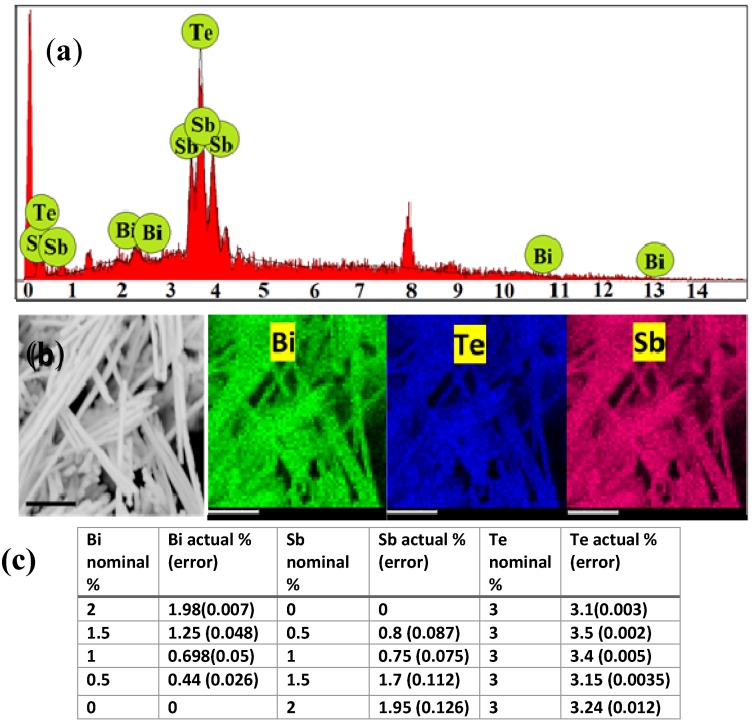
(**a**) Representative EDX spectrum of Bi_2−*x*_Sb*_x_*Te_3_ NWs; (**b**) Mapping of the Bi_2−*x*_Sb*_x_*Te_3_ NWs reveal uniform presence of the constituent elements: bismuth (green), tellurium (blue) and antimony (pink). The scale bar in the mapping indicates 1 µm; (**c**) Table showing the nominal atomic % of Bi, Sb and Te elements and the actual atomic % of the same elements present in the samples, which reveal that the samples are slightly rich in tellurium. Tellurium may be present in metallic form within the samples.

EDX analyses of all the samples were conducted in order to investigate the actual compositional variation within the NWs which are shown in Figure 2. In general, all the Bi_2−*x*_Sb*_x_*Te_3_ NWs did show presence of Bi, Sb and Te elements suggesting the formation of Bi_2−*x*_Sb*_x_*Te_3_ (Figure 2a). EDX mapping on the template-freed NWs also indicated uniform distribution of all the three elements in the samples revealing successful growth of Bi_2−*x*_Sb*_x_*Te_3_ alloy compositions. EDX analyses performed for each nominal composition of Bi_2−*x*_Sb*_x_*Te_3_ NWs as performed on over 50 different NWs indicated the actual at % of Bi and Sb elements present in each sample as shown in Figure 2c. It is to be noted that with increasing Bi/Sb ratio in the electrolyte (nominal wt %), the Bi/Sb ratio increased in the as-grown NWs (actual wt %) without any post-treatment such as annealing.

**Figure 3:**
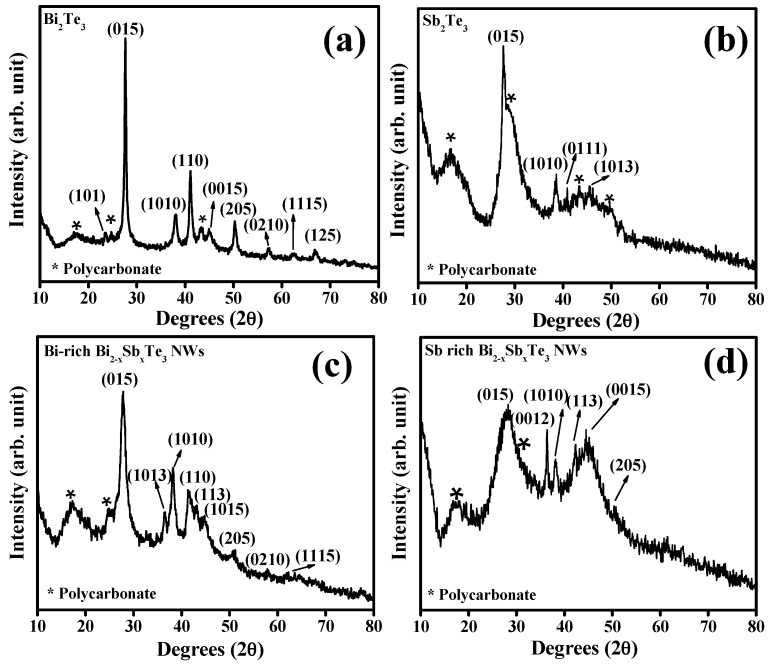
Representative XRD patterns of Bi_2−*x*_Sb*_x_*Te_3_ NWs of different nominal compositions; (**a**) for *x* = 0 i.e., pure Bi_2_Te_3_ (**b**) for *x* = 2 i.e., Sb_2_Te_3_ (**c**) Bi-rich and (**d**) Sb-rich Bi_2−*x*_Sb*_x_*Te_3_ NWs. The NWs show a noticeable peak broadening with increasing Sb, due to the grain size difference between the Bi-rich and Sb-rich compositions, showing polycrystalline signature for all of the types of NWS.

Figure 3 shows the XRD spectra for different compositions of Bi_2−*x*_Sb*_x_*Te_3_ NWs grown in PC templates. The sharp peak around 2θ = 28° corresponds to the (015) crystallographic direction and there were also relatively strong peaks observed at 2θ = 27° from (110) direction. As the Bi_2−x_Sb*_x_*Te_3_ NWs have relatively larger diameters (~200 nm), polycrystalline orientations were more prevalent as revealed from the XRD spectra, and are in agreement with other reports [28,29,38]. Contributions from the amorphous PC template also added to the background at lower angle 2θ between 10° and 50°. Several reports have focused on pore diameter as being one of the most influential factors in the physical properties of template-grown NWs [28,29,39]. It is to be noted that as the Sb content increased in the samples, the crystallinity in the sample was found to significantly decrease. While the peaks for pure Bi_2_Te_3_ NWs prepared under similar deposition conditions are relatively sharp indicating highly crystalline grain domains, alloy compositions rich in Sb show broad XRD peaks indicating either low crystallinity or small crystallite sizes commensurate with our SEM observation in Figure 1f. 

**Figure 4:**
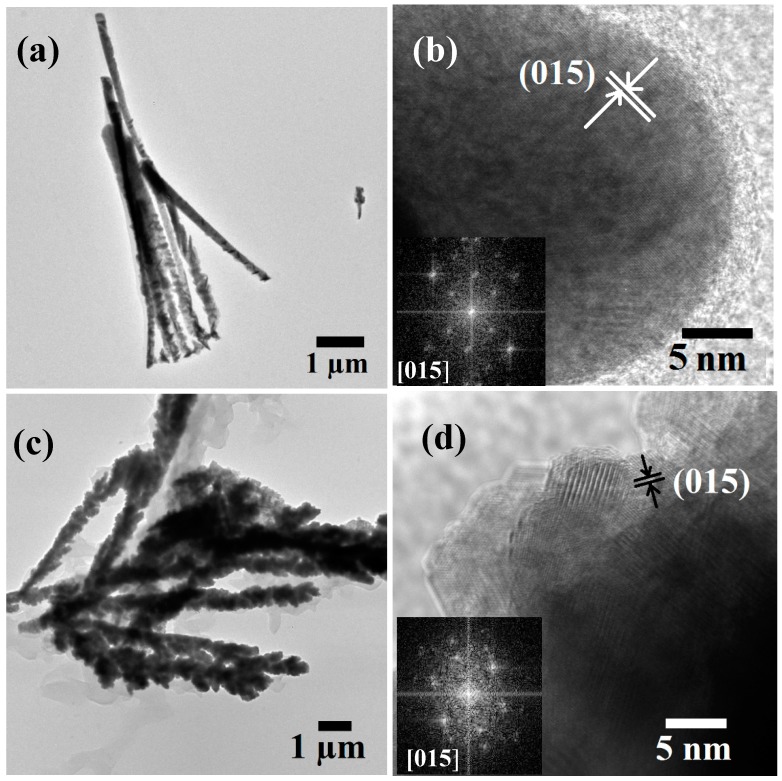
TEM and HRTEM images of (**a**,**b**) of Bi-rich Bi_2−*x*_Sb*_x_*Te_3_ NWs showing uniform and smooth surface texture composed of well crystalline spherical grains; (**c**,**d**) Sb-rich Bi_2−*x*_Sb*_x_*Te_3_ NWs with rough surface that are composed of highly crystalline faceted grains.

In order to understand the observed peak broadening with increasing Sb content in the NWs, TEM and HRTEM was carried out on both Bi-rich and Sb-rich Bi_2−*x*_Sb*_x_*Te_3_ compositions as shown in Figure 4. As expected Bi-rich Bi_2−*x*_Sb*_x_*Te_3_ NWs showed uniform thickness along the length due to the presence of large spherical particulate crystallites (Figure 4a,b). The polycrystalline NWs have a crystallite size of ~30 nm and show well-defined crystalline behavior with preferential development of (015) plane (*d* spacing ~0.32 nm). Fast Fourier Transform (FFT) patterns of the (015) lattice plane showed crystalline spots depicting plausible hexagonal crystal structure of the constituent nano-sized grains, corresponding to the rhombohedral crystal system of Bi_2−*x*_Sb*_x_*Te_3_ alloys. On the contrary, Sb-rich Bi_2−*x*_Sb*_x_*Te_3_ NWs have rough surfaces as shown in Figure 4c, due to the clear hexagonal/rhombohedral crystallites of ~5–10 nm in size. These nanocrystallites are also highly crystalline, and show major development of (015) planes. FFT patterns similarly showed rhombohedral disposition of the nanocrystallites. Hence the broadness of the XRD peaks in Sb-rich compositions can be accounted for due to small crystallite sizes in the polycrystalline NWs, and not due to low crystallinity. The formation of polycrystalline Bi_2−*x*_Sb*_x_*Te_3_ NWs can be explained by considering a competitive growth mechanism during the electrochemical growth of the Bi_2−*x*_Sb*_x_*Te_3_ NWs: (i) rapid nucleation on the conducting gold layer and (ii) nucleation followed by formation of new grains. While single crystalline NWs are expected with the formation of larger nuclei and subsequent fast growth along the template pores, polycrystalline NWs may form if small nuclei are formed at the first instance followed by slow and steady growth of the nuclei. The two processes compete with each other and depend on several parameters including the potential difference, nature of the electrodes from which the nucleation start, the particle size of the sputtered back electrode, as well as the electrolyte composition [40]. We argue that profuse availability of Bi ions from aqueous ionic Bi-nitrate during the growth of Bi-rich compositions might have led to rapid growth of the nuclei and hence larger crystallites. On the other hand, low availability of Sb ions due to slow release of Sb ions from the Sb-tartaric acid complex might cause a steady and slow growth of nuclei and hence this resulted in finer grain sizes in Sb-rich compositions [41]. While high temperature annealing might lead to enhanced crystallinity of the NWs, this could also potentially lead to the fractionalization of ternary alloy composition and formation of sub-oxide impurities leading to high resistivity in the sample. Therefore, our electrochemical growth process at room temperature is a step forward in growing the complex Bi_2−*x*_Sb*_x_*Te_3_ composition as pure and highly crystalline NWs.

**Figure 5:**
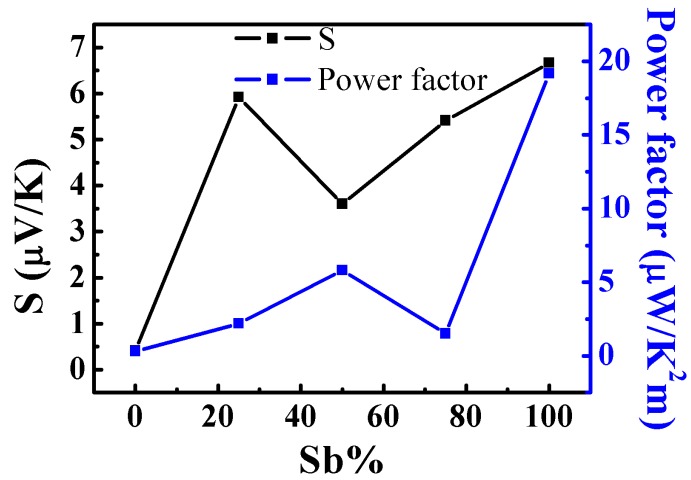
Relative Seebeck coefficient and power factor of the Bi_2−*x*_Sb*_x_*Te_3_ NWs plotted as a function of the nominal Sb content in the samples.

Our Bi_2−*x*_Sb*_x_*Te_3_ NWs encapsulated within a PC matrix may serve as a lightweight flexible TEG as can be demonstrated from the measured relative Seebeck coefficients of the above samples. For each measurement, NW-filled PC template were cut into pieces having cross-sectional area of ~0.15–0.20 cm^2^, and sputter-coated with Au electrodes on the top surface. The results of these measurements are depicted in Figure 5 (electrical conductivity data is shown in the Supporting Information). From Figure 5, we can clearly see that the relative Seebeck coefficient increased with increasing Sb content, peaking at 100 at % (nominal at %). The samples show p-type behavior over the entire composition range. The power factor, calculated as *S*^2^*σ*, is also shown in the figure. From these results we can see that although the power factor obtained is less than what has been reported in the literature [16,17,18,19] for Bi and Sb tellurides (see also Supporting Information), it is worth noting that this performance has been achieved by using a much cheaper processing technique, which utilises only a small amount of material, and the final product can be used in applications where flexibility of the devices is needed in order to conformally wrap them around complex-shaped objects. On the other hand, we also continue to improve our measurement procedure as sensing the precise temperature on either sides of such a thin sample is very challenging and potentially, can result in overestimation the temperature difference (and hence, underestimating the relative Seebeck coefficient and power factor). It should also be noted here that the thermoelectric properties of arrays of template-grown nanowires of Bi_2−*x*_Sb*_x_*Te_3_ still embedded in the template are rarely found in the literature. Most of the reported measurements relate to individual nanowires freed from the template. Such single nanowires exhibit better thermoelectric properties than that reported here because of fewer defects, better crystallinity and better preserved structure of the individual nanowire being tested (see Supporting Information). However, isolated nanowires would prove difficult to implement in actual thermoelectric device applications. Instead, in this work, we have presented measurements of the thermoelectric performance of embedded nanowire arrays which can be directly incorporated in real life applications. The thermoelectric parameters evaluated in this way are lower than the literature reports of single nanowires (see Supporting Information), due to higher probability of encountering a small fraction of defective nanowires containing imperfections such as anti-site defects, Te-rich nano-nuggets, accidental Au doped regions, etc., which affect the Seebeck coefficient and thermoelectric power factor [42,43,44,45,46,47,48,49]. Nevertheless, evaluation of thermoelectric performance of the as-grown template-embedded nanowires offers valuable insight into their applicability in thermoelectric devices.

## 4. Conclusions

In conclusion, we have successfully prepared Bi_2−*x*_Sb*_x_*Te_3_ NWs of different compositions with varying Sb/Bi ratios (both Bi-rich and Sb-rich) in flexible PC templates, via a one-step room temperature electrodeposition process. The process involves simple tuning of tartaric acid and nitric acid electrolyte composition at an optimized voltage condition of −0.5 V between the two electrodes for ~90 min. The structure and crystallinity of the NWs studied by SEM, XRD and TEM indicates that the NWs are polycrystalline with high order of crystallinity. NWs of Sb-rich compositions formed small crystallites with dimensions of the order of 5 nm, as compared to ~30 nm in Bi-rich compositions, possibly due to the difference in the rate of growth after nucleation as a result of slow dissociation of Sb ions from tartaric acid complex. We measured TE properties of our as-grown NWs using a bespoke thermoelectric set-up. Our high crystalline ternary Bi_2−*x*_Sb*_x_*Te_3_ NWs encapsulated within flexible and lightweight PC matrix showed reasonably good TE properties with Sb_2_Te_3_ NWs sample showing a relative Seebeck coefficient of ~6.5 μV/K.

## Supplementary Materials

The following are available online at www.mdpi.com/1996-1944/10/5/553/s1. Figure S1: (a) Large crystals grown on top of the template surface blocking the pores in uncleaned template during deposition and (b) Unfilled template with incomplete NW growth as a result as shown in the cross section; Figure S2: (a) A representative sample used for characterization; (b) Peltier module used to heat or cool the sample surface; (c) the Peltier module-copper block assembly used to heat/cool top or bottom surfaces of the sample.

## References

[1] Bell L.E. (2008). Cooling, heating, generating power, and recovering waste heat with thermoelectric systems. Science.

[2] Tritt T.M., Subramanian M. (2006). Thermoelectric materials, phenomena and applications: A bird’s eye view. MRS Bull..

[3] Rosenberg Y., Gelbstein Y., Dariel M.P. (2012). Phase separation and thermoelectric properties of the Pb_0.25_Sn_0.25_Ge_0.5_Te compound. J. Alloys Compd..

[4] Gelbstein Y., Dashevsky Z., Dariel M.P. (2007). In-doped Pb_0.5_Sn_0.5_Te p-type samples prepared by powder metallurgical processing for thermoelectric applications. Physica B.

[5] Gelbstein Y., Davidow J. (2014). Highly efficient functional Ge_*x*_Pb_1−x_Te based thermoelectric alloys. Phys. Chem. Chem. Phys..

[6] Gelbstein Y. (2013). Phase morphology effects on the thermoelectric properties of Pb_0.25_Sn_0.25_Ge_0.5_Te. Acta Mater..

[7] Kirievsky K., Shlimovich M., Fuks D., Gelbstein Y. (2014). An ab initio study of the thermoelectric enhancement potential in nano-grained TiNiSn. Phys. Chem. Chem. Phys..

[8] Kirievsky K., Gelbstein Y., Fuks D. (2013). Phase separation and antisite defects in the thermoelectric TiNiSn half-Heusler alloys. J. Solid State Chem..

[9] Sadia Y., Dinnerman L., Gelbstein Y. (2013). Mechanical Alloying and Spark Plasma Sintering of Higher Manganese Silicides for Thermoelectric Applications. J. Elect. Mater..

[10] Gelbstein Y., Tunbridge J., Dixon R., Reece M.J., Ning H.P., Gilchrist R., Summers R., Agote I., Lagos M.A., Simpson K. (2014). Physical, Mechanical, and Structural Properties of Highly Efficient Nanostructured n- and p-Silicides for Practical Thermoelectric Applications. J. Elect. Mater..

[11] Hicks L.D., Dresselhaus M.S. (1993). Effect of quantum-well structures on the thermoelectric figure of merit. Phys. Rev. B.

[12] Dresselhaus M.S., Chen G., Tang M.Y., Yang R., Lee H., Wang D., Ren Z., Fleurial J.P., Gogna P. (2007). New Directions for Low-Dimensional Thermoelectric Materials. Adv. Mater..

[13] Heremans J.P., Thrush C.M., Morelli D.T. (2005). Thermopower enhancement in PbTe with Pb precipitates. J. Appl. Phys..

[14] Goldsmid H.J. (1964). Thermoelectric Refrigeration.

[15] Yim W.M., Rosi F.D. (1972). Compound tellurides and their alloys for Peltier cooling—A review. Solid-State Electron..

[16] Datta A., Paul J., Kar A., Patra A., Sun Z., Chen L., Martin J., Nolas G.S. (2010). Facile Chemical Synthesis of Nanocrystalline Thermoelectric Alloys Based on Bi-Sb-Te-Se. Cryst. Growth Des..

[17] Finefrock S.W., Yang H., Fang H., Wu Y. (2015). Thrmoelectric Properties of Solution Synthesized Nanostructured Materials. Annu. Rev. Chem. Biomol. Eng..

[18] Zheng G., Su X., Li X., Liang T., Xie H., She X., Yan Y., Uher C., Kanatzidis M.G., Tang X. (2016). Toward High-Thermoelectric-Performance Large-Size Nanostructured BiSbTe Alloys via Optimization of Sintering-Temperature Distribution. Adv. Energy Mater..

[19] Xie W., Tang X., Yan Y., Zhang Q., Tritt T.M. (2009). High thermoelectric performance BiSbTe alloy with unique low-dimensional structure. J. Appl. Phys..

[20] Popescu A., Woods L.M. (2010). Enhanced thermoelectricity in composites by electronic structure modifications and nanostructuring. Appl. Phys. Lett..

[21] Minnich A.J., Dresselhaus M.S., Ren Z.F., Chen G. (2009). Bulk nanostructured thermoelectric materials: Current research and future prospects. Energy Environ. Sci..

[22] Chen Z.G., Han G., Yang L., Cheng L., Zou J. (2012). Nanostructured thermoelectric materials: Current research and future challenge. Prog. Nat. Sci. Mater. Int..

[23] Datta A., Popescu A., Woods L., Nolas G.S., Rowe D.M. (2012). Chapter 14: The Bottom-Up Approach to Bulk Thermoelectric Materials with Nanoscale Domains in Materials, Preparation, and Characterization. Thermoelectrics in Materials, Preparation, and Characterization in Thermoelectrics.

[24] Prieto A.L., Sander M.S., Gonzalez M.M., Gronsky R., Sands T., Stacy A.M. (2001). Electrodeposition of Ordered Bi_2_Te_3_ Nanowire Arrays. J. Am. Chem. Soc..

[25] Gonzalez M.M., Snyder G.J., Prieto A.L., Gronsky R., Sands T., Stacy A.M. (2003). Direct Electrodeposition of Highly Dense 50 nm Bi_2_Te_3−*y*_Se_*y*_ Nanowire Arrays. Nano Lett..

[26] Xiao F., Yoo B.Y., Lee K.H., Myung N.S.V. (2007). Electro-transport studies of electrodeposited (Bi_1−*x*_Sb*_x_*)_2_Te_3_ nanowires. Nanotechnology.

[27] Zhang G., Yu Q., Li X. (2010). Wet chemical synthesis and thermoelectric properties of V-VI one- and two-dimensional nanostructures. Dalton Trans..

[28] Frantz C., Stein N., Zhang Y., Bouzy E., Picht O., Toimil-Molares M.E., Boulanger C. (2012). Electrodeposition of bismuth telluride nanowires with controlled composition in polycarbonate membranes. Electrochim. Acta.

[29] Picht O., Mueller S., Alber I., Rauber M., Falk J.L., Medlin D.L., Neumann R., T-Molares M.E. (2012). Tuning the Geometrical and Crystallographic Characteristics of Bi_2_Te_3_ Nanowires by Electrodeposition in Ion-Track Membranes. J. Phys. Chem. C.

[30] Albrecht T.T., Schotter J., Kastle G.A., Emley N., Shibauchi T., Elbaum L.K., Guarine G., Black C.T., Touminen M.T., Russell T.P. (2000). Ultrahigh-density nanowire arrays grown in self-assembled diblock copolymer templates. Science.

[31] Bal M., Ursache A., Tuominen M.T., Goldbach J.T., Russell T.P. (2002). Nanofabrication of integrated magnetoelectronic devices using patterned self-assembled copolymer templates. Appl. Phys. Lett..

[32] Martin C.R. (1994). Nanomaterials: A membrane-based synthetic approach. Science.

[33] Hariri M.B., Dolati A., Moakhar R.S. (2013). The Potentiostatic Electrodeposition of Gold Nanowire/Nanotube in HAuCl4 Solutions Based on the Model of Recessed Cylindrical Ultramicroelectrode Array. J. Electrochem. Soc..

[34] Boughey F.L., Davies T., Datta A., Whiter R.A., Sahonta S.L., Kar-Narayan S. (2016). Vertically aligned zinc oxide nanowires electrodeposited within porous polycarbonate templates for vibrational energy harvesting. Nanotechnology.

[35] Ou C., Sanchez-Jimenez P.E., Datta A., Boughey F.L., Whiter R.A., Sahonta S.L., Kar-Narayan S. (2016). Template-Assisted Hydrothermal Growth of Aligned Zinc Oxide Nanowires for Piezoelectric Energy Harvesting Applications. ACS Appl. Mater. Interfaces.

[36] Smith M., Choi Y.S., Boughey C., Kar-Narayan S. (2017). Controlling and assessing the quality of aerosol jet printed features for large area and flexible electronics. Flex. Print. Electron..

[37] Martin-Gonzalez M.S., Prieto A.L., Gronsky R., Sands T., Stacy A.M. (2002). Insights into the Electrodeposition of Bi_2_Te_3_. J. Electrochem. Soc..

[38] Chang T., Cho S., Kim J., Schoenleber J., Frantz C., Stein N., Boulanger C., Lee W. (2015). Individual thermoelectric properties of electrodeposited bismuth telluride nanowires in polycarbonate membranes. Electrochim. Acta.

[39] Sander M.S., Gronsky R., Sands T., Stacy A.M. (2003). Structure of Bismuth Telluride Nanowire Arrays Fabricated by Electrodeposition into Porous Anodic Alumina Templates. Chem. Mater..

[40] Budevski E., Staikov G., Lorenz W.J. (1996). Electrochemical Phase Formation and Growth.

[41] Bouroushian M., Schloz F. (2010). Electrochemistry of Metal Chalcogenides. Monographs in Electrochemistry.

[42] Pinisetty D., Davis D., Podlaha-Murphy E.J., Murphy M.C., Karki A.B., Young D.P., Devireddy R.V. (2011). Fabrication and characterization of electrodeposited antimony telluride crystalline nanowires and nanotubes. J. Mater. Chem..

[43] Chen C.L., Chen Y.Y., Lin S.J., Ho J.C., Lee P.C., Chen C.D., Harutyunyan S.R. (2010). Fabrication and Characterization of Electrodeposited Bismuth Telluride Films and Nanowires. J. Phys. Chem. C.

[44] Xu E., Li Z., Acosta J.A., Li N., Swartzentruber B., Zheng S.J., Sinitsyn N., Htoon H., Wang J., Zhang S. (2016). Enhanced thermoelectric properties of topological crystalline insulator PbSnTe nanowires grown by vapor transport. Nano Res..

[45] Kumar P., Pfeffer M., Peranio N., Eibl O., Bäßler S., Reith H., Nielsch K. (2017). Ternary, single-crystalline Bi_2_ (Te, Se)_3_ nanowires grown by electrodeposition. Acta Mater..

[46] Lee P., Chen H., Tseng C., Lee C., Chang C., Chen Y. (2013). Thermoelectric Properties of an Individual Bi_1.75_Sb_0.25_Te_2.02_ Nanowire. Chin. J. Phys..

[47] Bäßler S., Böhnert T., Gooth J., Schumacher C., Pippel E., Nielsch K. (2013). Thermoelectric power factor of ternary single-crystalline Sb_2_Te_3_- and Bi_2_Te_3_-based nanowires. Nanotechnology.

[48] Mannam R.S., Davis D. (2010). High Seebeck Coefficient BiSbTe Nanowires. Electrochem. Solid-State Lett..

[49] Ruoho M., Juntunen T., Tittonen I. (2016). Large-area thermoelectric high-aspect-ratio nanostructures by atomic layer deposition. Nanotechnology.

