# Supplementary material for: Structure and Thermoelectric Properties of Bi2−xSbxTe3 Nanowires Grown in Flexible Nanoporous Polycarbonate Templates
Source: Materials (Basel). 2017 May 19;10(5):553. doi: 10.3390/ma10050553 (PMC5459006; doi:10.3390/ma10050553)
Supplement: Supplementary file 1 [file materials-10-00553-s001.pdf]

Supplementary Materials: Structure and Thermoelectric Properties of $Bi_{2-x}Sb_xTe_3$ Nanowires Grown in Flexible Nanoporous Polycarbonate Templates

Anuja Datta, Abhijeet Sangle, Nick Hardingham, Charles Cooper, Max Kraan, David Ritchie, Vijay Narayan and Sohini Kar-Narayan

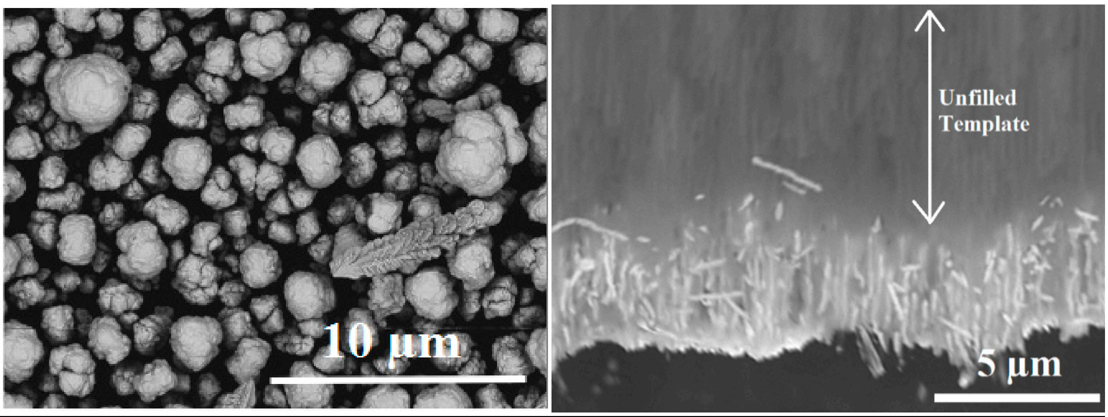

Figure S1: (**a**) Large crystals grown on top of the template surface blocking the pores in uncleaned template during deposition and (**b**) Unfilled template with incomplete NW growth as a result as shown in the cross section.

Thermoelectric Measurement Setup:

The thermoelectric measurement setup used for characterization of seebeck co-efficient is shown in figure S1. As shown in figure S1a, four enameled conducting Cu wires were attached to the top and bottom of the sample using Ag paint. The sample was placed between two commercial Peltier modules which were used to heat/cool the top or bottom surfaces of the sample by sandwiching the sample between them, as indicated by yellow broken arrow in figure S1a,c. The temperature of the Peltier modules was monitored by using a resistance thermometer (either Ag, printed by using an aerosol-jet printer or a thin Pd sputter-coated film), as shown in Figure S1b. (The black line covered in kapton protecting tape is a thin Pd film). The top half of the assembly (see figure S1c) was mounted on a rack and pinion mechanism which allowed it moving up and down, which allowed to place the sample and apply a temperature gradient by sandwiching the sample between the top and bottom Peltier modules. Copper blocks were attached to the Peltier modules on the sides opposite to those facing the sample. These blocks functioned as heat sinks and ensured that the temperature of the Peltier face in direct contact with them remained relatively constant, which in turn ensured that the temperature of the other Peltier face in contact with the sample also remained fairly constant, through a constant DC bias applied to power the Peltier modules. The DC bias applied to the Peltier modules was varied to vary the temperature gradient applied across the sample. It was assumed that the mean temperature of the sample did not change much, as the Peltier modules were heated/cooled relative to the Cu blocks placed at room temperature. The entire assembly was put inside an enclosure, which had N_2 gas constantly flowing, to ensure uniformity of temperature in the surrounds of the Peltier modules and Cu blocks attached to them, and to suppress the condensation of ambient water vapour on the cold Peltier module during the measurement.

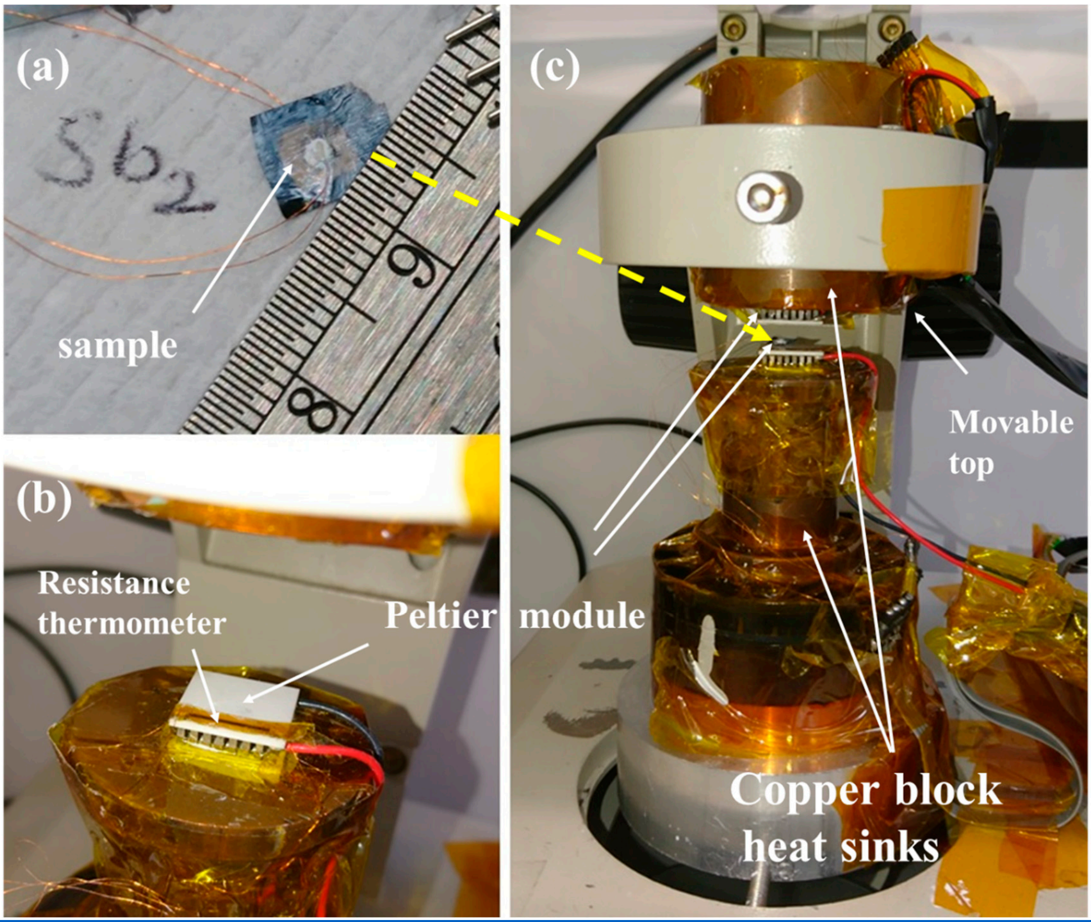

Figure S2: (**a**) A representative sample used for characterization; (**b**) Peltier module used to heat or cool the sample surface; (**c**) the Peltier module-copper block assembly used to heat/cool top or bottom surfaces of the sample.

© 2017 by the authors. Submitted for possible open access publication under the terms and conditions of the Creative Commons Attribution (CC BY) license (http://creativecommons.org/licenses/by/4.0/).
